# Supplementary material for: The Impact of Perioperative Fluid Balance on Postoperative Complications after Esophagectomy for Esophageal Cancer
Source: J Clin Med. 2022 Jun 5;11(11):3219. doi: 10.3390/jcm11113219 (PMC9181193; doi:10.3390/jcm11113219)
Supplement: Supplementary file 1 [file jcm-11-03219-s001.zip › jcm-1738718-supplementary.pdf]

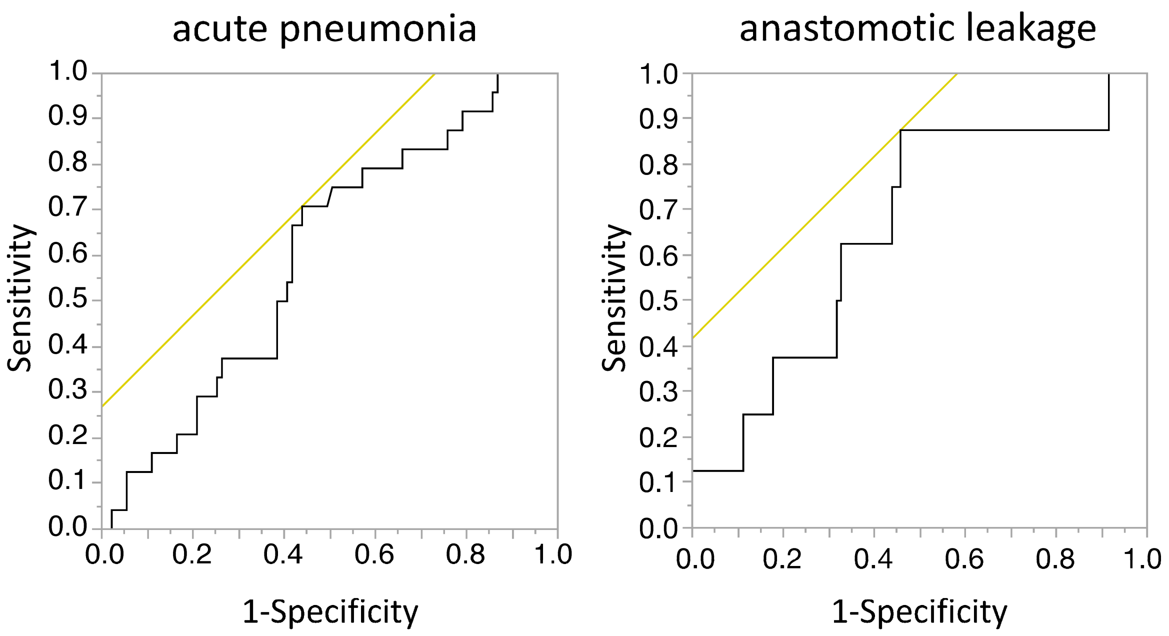

Figure S1. Cutoff values using ROC curves for the fluid balance at POD1. The fluid balance cut off values of postoperative acute pneumonia and anastomotic leakage were 3080 mL and 3094 mL, respectively (acute pneumonia; sensitivity of 0.71 and specificity of 0.44, anastomotic leakage; sensitivity of 0.88 and specificity of 0.46).
